# Cataract surgery outcomes: comparison of the extracapsular cataract extraction and manual small incision cataract surgery techniques

**DOI:** 10.4314/ahs.v22i1.72

**Published:** 2022-03

**Authors:** Amukelani Jimmy Zitha, Nishanee Rampersad

**Affiliations:** 1 Themba Hospital, Mpumalanga Province, Private Bag X1002, Kabokweni, 1245 South Africa; 2 Discipline of Optometry, School of Health Sciences, University of KwaZulu-Natal, Private Bag X54001, Durban, 4000 South Africa

**Keywords:** Cataract surgery outcomes, extracapsular cataract surgery, manual small incision cataract surgery, ocular complications

## Abstract

**Background:**

Blindness and visual impairment are public health problems and constitute an important socio-economic burden in sub-Saharan Africa. Understanding the outcomes of cataract surgery will improve our knowledge of risk factors for poor outcomes. Previous studies have focused exclusively on the phacoemulsification technique with limited attention to the extracapsular cataract extraction (ECCE) and manual small incision cataract surgery (MSICS) techniques.

**Objectives:**

To compare the cataract surgery outcomes between the ECCE and MSICS techniques.

**Methods:**

The study was an observational research design that used the LogMAR visual acuity (VA) chart, subjective refraction, slit lamp and ophthalmoscope to collect data. The participants were followed for a period of six-weeks post-surgery and outcomes were recorded. Data were presented using frequencies, percentages and means ± standard deviation.

**Results:**

The sample included 101 participants, with a mean age of 66.32 ± 15.99 years. Fifty and 51 participants had undergone the ECCE and MSICS techniques respectively. Overall, one-hundred participants had poor pre-surgery VA and subjective refractions were generally not possible due to the severity of cataracts present. The mean aided post-surgery VA was 0.31 LogMAR and 0.13 LogMAR in the ECCE and MSICS groups respectively (p < 0.001). The mean post-surgery refractive astigmatism was similar in the ECCE (-2.06 D) and MSICS (-1.80 D) groups (p = 0.110). The spherical equivalence was approximately -0.50 D higher in the MSICS group, but not statistically significant (p = 0.330). Approximately one out of every five participants (n = 21) had post-surgery ocular complications such as corneal opacity and haziness as well as posterior capsular absence.

**Conclusions:**

The MSICS technique showed better post-surgery outcomes than the ECCE technique.

## Introduction

Cataract, which is the opacification of the crystalline lens in the eye, causes a gradual decrease in vision, and can eventually lead to blindness.^1^,^2^ Blindness and visual impairment (VI) are public health problems and constitute an important socio-economic burden in sub-Saharan Africa.^3^ Cataract is the leading cause of global blindness and accounts for half of the blindness in Africa.^4^,^5^,^6^,^7^,^8^,^9^ Cataract is usually more prevalent in adults but may occur as a congenital disorder in children.^10^,^11^,^12^ Age-related cataract is the most common and often presents as a bilateral yet asymmetrical condition.^4^ The most common risk factors known to cause cataract include smoking, diabetes and exposure to ultraviolet light while other risk factors include trauma, uveitis, excessive alcohol consumption, high body mass index and hypertension.^1^,^2^,^4^,^13^

Surgery is an effective treatment for cataract and involves removing the cloudy crystalline lens and replacing it with an intraocular lens (IOL).^14^,^15^ Common cataract surgery techniques used in developing countries include the extracapsular cataract extraction (ECCE) and manual small incision cataract surgery (MSICS) techniques.^7^,^16^,^17^ These techniques use local anaesthetic but general anaesthesia is indicated if an individual is unable to lie still owing to communication and/or physical factors. In both the ECCE and MSICS techniques incisions are made in the anterior chamber capsule through which the crystalline lens nucleus is removed. An IOL, which is made from hard plastic (polymethylmethacrylate), is inserted and anchored by the capsule. The wound is sutured in the ECCE technique and left unsutured in the MSICS technique implying a self-sealing wound.^17^,^18^

The assessment of post-surgery outcomes is important for the detection, management and monitoring of VI, refractive error and post-surgery ocular complications. Methods for evaluating outcomes include measurements of visual acuity (VA), subjective refraction and ophthalmoscopy. 14,21,22,23 Understanding the influence of the outcomes for the two techniques can improve our understanding of the possible mechanisms and risk factors for poor outcomes.^19^, ^24^,^25^,^26^,^27^,^28^,^29^ Therefore, the objective of this study was to compare the cataract surgery outcomes between the ECCE and MSICS techniques at a hospital in the Mpumalanga province of South Africa.

## Methods

This study (reference number BE592/16) obtained ethical approval from the Biomedical Research and Ethics Committee of the University of KwaZulu-Natal and followed the tenets of the Declaration of Helsinki. Site approval was attained from the Mpumalanga Department of Health and the Themba Hospital Chief Executive Officer. All participants provided written informed consent after a discussion of the nature and procedures involved in the study. The study employed an observational research design and was conducted at the Themba Hospital. Convenience sampling was used to recruit 101 participants including African and Mixed race populations.

The VA and refractive status were assessed using the LogMAR VA chart and subjective refraction respectively. 21,32,33 The spherical equivalence (SE) was calculated as the sphere power plus half the negative cylinder power to constitute the final prescription.^30^,^31^ Slit lamp biomicroscopy (Takagi MS-70N) and ophthalmoscopy were used to assess the anterior and posterior segments respectively. To ensure standardisation, the cataract surgery outcomes were assessed by only one researcher.

Data were captured and analysed with the Statistical Package for Social Sciences (SPSS) software (version 25). Overall, the data were summarised using frequencies, percentages and means ± standard deviation. The independent t-test was used to assess the differences in demographic (age) and visual characteristics (VA and refractive error) between the two cataract surgery groups. The study adopted a 95% significance level where p-values less than 0.05 were considered statistically significant.

## Results

### Demographic characteristics

The study sample included 101 participants (52 females and 49 males) who had undergone either the ECCE or MSICS techniques. The study sample consisted of majority African participants compared with Mixed race participants (100 versus 1) aged between 9 and 94 years with a mean of 66.32 ± 15.99 years. The age groups from 21 to 40 years and 61 to 80 years consisted of the lowest (3%) and highest (64%) percentages respectively. The mean ages for the ECCE (69.66 ± 14.55 years) and MSICS (63.04 ± 16.79 years) groups show that participants who had the ECCE technique were significant older (p = 0.037) but the difference may not be clinically significant as it was only six years.

### Visual acuity characteristics

**Figure 1:**
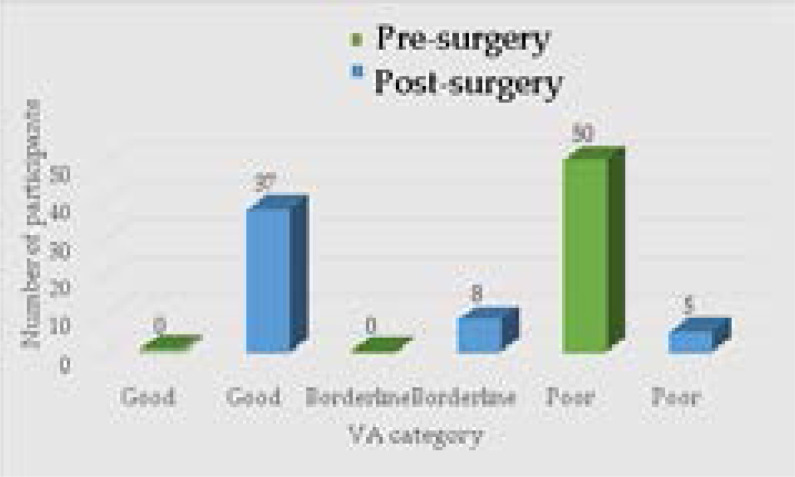
Frequency of aided VA categories for the ECCE (n = 50) group pre-surgery and post-surgery

The pre-surgery and post-surgery aided VA, which was assessed and recorded at six-week follow up, were categorised as either poor (>1.00 LogMAR), borderline (1.00 – 0.60 LogMAR), or good (0.48 – 0.00 LogMAR).19 All participants in the ECCE group (n = 50) presented with poor aided pre-surgery VA (Figure 1). However, 37 participants in the ECCE group showed good aided post-surgery VA while eight and five presented with borderline and poor aided post-surgery VA respectively (Figure 1). For the ECCE group, the mean aided post-surgery VA was 0.31 LogMAR.

**Figure 2:**
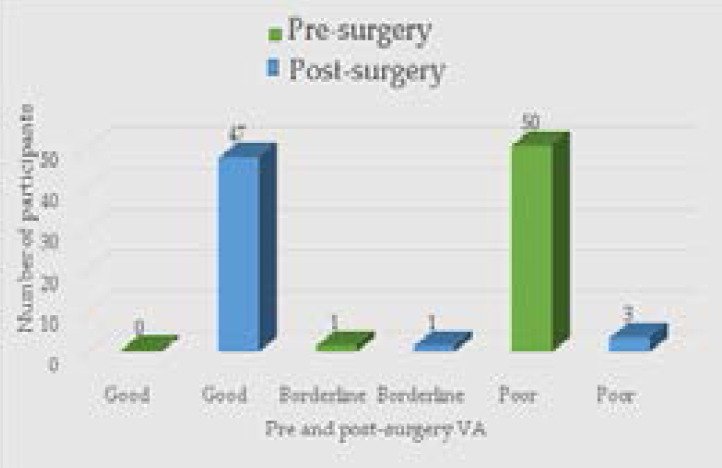
Frequency of aided VA categories for the MSICS (n = 51) Group pre-surgery and post-surgery

For the MSICS group, 47 participants had good aided post-surgery VA while one participant each had borderline VA pre-surgery and post-surgery (Figure 2). The mean post-surgery aided VA was 0.13 LogMAR which was better than that of the ECCE technique and was statistically significant (p < 0.001).

### Refraction characteristics

A subjective refraction was performed at the six-week follow up post-surgery. For all the participants (n = 101), pre-surgery refraction was not possible due to the density of the cataract. In total, 43 and 49 participants in the ECCE and MSICS groups respectively presented with post-surgery refractive astigmatism. Furthermore, 29 and 34 participants in the ECCE and MSICS groups presented with against-the-rule (ATR) astigmatism while two and five participants had with-the-rule (WTR) astigmatism in the ECCE and MSICS groups respectively. Twelve and 10 participants presented with oblique astigmatism in the ECCE and MSICS groups respectively while seven and two participants in the ECCE and MSICS groups presented with spherical refractive error.

Overall, the sphere power ranged from -3.75 D to +12.50 D and -3.00 D to +1.25 D in the ECCE and MSICS groups respectively. The mean sphere power was low in the ECCE group (-0.09 D) compared with the MSICS group (-0.67 D) although this difference was not statistically significant (p = 0.259). The cylinder power ranged from -3.75 D to -0.75 D and -3.50 D to -0.25 D for the ECCE and MSICS groups respctively. The mean cylinder power was low in the MSICS group (-1.80 D) compared with the ECCE group (-2.06 D) although the difference was not statistically significant (p = 0.110) (Table 1). The SE refraction ranged from -4.75 D to +10.75 D and -3.75 D to +0.50 D for the ECCE and MSICS groups respectively. The SE refraction was approximately -0.50 D higher in the MSICS group. However, this difference was small and not statistically significant (p = 0.330).

**Table 1:** Means and standard deviations for refraction characteristics of study participants (n = 101) at six-week follow up

Rx Characteristics	ECCE	MSICS
**Post-surgery Rx sphere (D)**	-0.09 ± 3.29	-0.67 ± 1.02
**Post-surgery Rx cylinder (D)**	-2.06 ± 0.76	-1.80 ± 0.77
**Post-surgery SE (D)**	-1.04 ± 3.35	-1.56 ± 1.03

Rx; Prescription, ECCE; Extracapsular cataract surgery, MSICS; Manual small incision cataract surgery, D; Diopters, SE; Spherical equivalence

### Ocular complications

Overall, 21 participants had ocular complications post-surgery (Table 2). For the ECCE group, there were 14 and two anterior and posterior segments post-surgery ocular complications respectively. For the MSICS group, the anterior and posterior segments ocular complications were noted with frequencies of one and four respectively.

**Table 2:** Frequency of anterior and posterior segment ocular complications for study participants at six-week follow up

Post- surgery ocular complications	Total (n =101)	ECCE (n = 50)	MSICS (n = 51)
**Anterior segment ocular complications**			
Hyphaema	1	1	0
Corneal opacity	3	3	0
Corneal scarring	1	1	0
Corneal haziness	4	4	0
Keratic precipitates	1	1	0
Iridodialysis	1	0	1
Iris prolapse	1	1	0
Others	3	3	0
**Posterior segment ocular complications**			
Neovascularisation	1	0	1
Vitreous opacity	1	0	1
Posterior capsular rupture	1	0	1
Posterior capsular absence	3	2	1

ECCE; Extracapsular cataract extraction, MSICS; Manual small incision cataract surgery

Overall, there were more anterior than posterior segment ocular complications post-surgery which were more commonly observed in the ECCE group. The cornea was the most common anterior segment structure to show post-surgery ocular complications including corneal opacity and haziness that were observed in three and four participants respectively in the ECCE group. Other anterior segment ocular complications, which included foreign body sensation, shallow anterior chamber and subconjunctival hemorrhage, were observed in the ECCE group (Table 2). One participant in the ECCE and MSICS groups presented with iris prolapse and iridodialysis respectively (Table 2). Overall, few posterior segment ocular complications as one participant in the MSICS group presented with vitreous opacity, neovascularisation, posterior capsular rupture and/or posterior capsular absence post-surgery.

## Discussion

### Visual acuity

This study set out to compare post-surgery outcomes between the ECCE and MSICS techniques. The study participants (n = 101) had undergone either the ECCE or MSICS techniques and consisted of majority older participants with a mean age of ∼66 ± 15.99 years including 49 male and 52 female participants. This finding is not surprising as age-related cataracts are commonly observed in older individuals. Furthermore the sample consisted of a similar proportion of male and female participants are most reported in the literature.^4^,^29^,^34^

A six-week follow up period in this study was considered adequate to allow for resolution and correction of any refractive error induced during the surgery.^34^ Some studies have used slightly longer post-surgery follow up periods of six to eight-weeks and they tend to show better post-surgery VA outcome.^25^,^34^,^36^,^38^ One-hundred participants presented with poor pre-surgery VA which is in agreement with the literature where the majority of study participants pre-surgery VA was poor.^36^,^37^ As expected, the VA in the two groups was better post-surgery as the purpose of cataract surgery is to improve and restore an individual's social, psycho-social and visual functions.^33^,^42^,^43^ In this study, 84 participants showed good post-surgery VA with best vision correction which is in accordance to the WHO standards.^34^,^39^,^40^,^41^ Overall, 47 and 37 had good post-surgery VA in the ECCE and MSICS groups respectively which compare well with results seen in other studies.^19^,^25^,^26^,^44^ The MSICS group achieved statistically better mean post-surgery aided VA. Despite reaching statistical significance, the mean post-surgery aided VA in the two groups would still be classified as good and therefore would also be a clinically significant improvement of post-surgery aided VA for the two groups.

The exact reason for the post-surgery VA variation between the two groups is unclear as there is no evidence of a difference in risks between the techniques.^45^ Poor post-surgery VA may result in the ECCE group owing to wound dehiscence due to a higher incidence of severe intraocular injury including choroidal haemorrhage, uveal and vitreous prolapse as well as retinal detachment.^46^,^47^,^48^,^49^ Furthermore, poor pre-surgery case selection and post-surgery uncorrected refractive error may result in poor post-surgery VA.^50^,^51^ Consequently, the MSICS technique is said to provide better post-surgery VA as small incision wounds are relatively resistant to trauma and the latter resistance differences may account for the post-surgery VA variations observed in the two groups.^46^,^47^ It is possible that the size of the smaller incision used for the MSICS technique was well as the overall surgical procedural differences may account for the variations noted in the post-surgery aided VA of the two groups.^16^,^17^

### Refraction characteristics

Ninety-two participants presented with post-surgery refractive astigmatism of moderate magnitude. The presence and amount of refractive astigmatism post-surgery is an important determining factor for visual outcome.27 Post-surgery, there was a slightly lower mean in the MSICS group compared with the ECCE group (-1.80 D versus -2.06 D). However, the difference was not statistically significant (p = 0.110). This observation of low post-surgery refractive astigmatism in the MSICS group is in agreement with the literature.^24^,^25^,^27^,^52^

Furthermore, ATR astigmatism was the most common type of post-surgery refractive astigmatism observed in 63 participants, while seven and 22 had oblique and WTR astigmatism respectively. This observation of a high prevalence of ATR astigmatism is in agreement with the literature. 27,45,53,54 A slightly higher mean sphere power was observed in the MSICS group compared with theECCE group (-0.67 D versus -0.09 D) but the difference was not statistically significant (p = 0.259).

Several studies suggest that cataract surgery leads to an increase in post-surgery refractive astigmatism because of the loss of tension in the sutures and consequently instability of corneal astigmatism that serve to influence the refractive changes in the eye.^44^,^53^,^55^,^56^,^57^,^59^,^60^,^61^,^62^ A change in the corneal curvature and tightly sewn sutures particularly with larger incision size are responsible for post-surgery refractive astigmatism.^44^,^56^,^60^,^61^,^62^ Furthermore, it is also possible that inappropriate biometry formula and customisation of A-constants may also increase systematic post-surgery refractive astigmatism.^58^,^59^

Post-surgery, individuals expect clear and optimal vision and less dependence on spectacle correction and this may be attained by reducing post-surgery refractive error with the use and refinement of the MSICS technique.60 Post-surgery, uncorrected refractive error results in blurred vision and glare as well as poor outcomes which is a problem particularly/span>in developing countries.^53^,^60^ In this study, the post-surgery mean SE for the ECCE and MSICS groups were less than 2.00 D. The post-surgery mean SE was lower in the ECCE group compared with the MSICS group (-1.04 D versus -1.56 D), but the difference was not statistically significant (p = 0.330). This observation is in accordance with the results observed in previous studies.^31^,^44^ Another study suggests that the increase in post-surgery mean SE may be due to the presence of myopia after cataract surgery.^63^

### Ocular complications

Overall, there was 16 and five ocular complications in the ECCE and MSICS groups respectively. The high prevalence of ocular complications in the ECCE group may be due to a large non-sealing incision after suturing of the wound as incision preparation is less traumatising to the trabeculum during the MSICS technique compared with the corneo-scleral incision with subsequent suturing during the ECCE technique.^27^,^35^,^67^ The MSICS technique produces a more closed procedure, owing to the self-sealing wound, where the risk of post-surgery ocular complications may be lower.^44^

The most common post-surgery anterior segment ocular complications were observed in the cornea including opacities (n = 3) and haziness (n = 4) in the ECCE group. Contradictory findings where corneal haziness was more prevalent in the MSICS group were reported in the literature. 19,20,25,27 Post-surgery corneal oedema affect the corneal endothelial cells which are essential for a transparent cornea as any alteration in the corneal endothelial cells is likely to result in corneal haziness, scarring and painful corneal bullae.^66^ Other post-surgery anterior segment ocular complications observed in this study included foreign body sensation, shallow anterior chamber, hyphaema and subconjunctival haemorrhage and such complications have been reported in previous studies.^19^,^27^,^34^,^35^ A shallow anterior chamber which may be seen if proper wound integrity is not maintained was observed in only one participant. 67 The presence of hyphaema was observed in only one participant in the ECCE group and may be due to focal vascularisation of the wound created during surgery.^68^

In developing countries, post-surgery ocular complications are major causes of VI.^34^,^50^,^51^ In this study, posterior segment ocular complications including posterior capsular rupture, vitreous opacity and neovascularisation were observed in the MSICS group while posterior capsular absence and/or rupture was observed in both groups. It has been suggested that trauma during insertion of IOL and puncture from loose canulas in the hydration process are likely to result in posterior capsular rupture.^69^ Furthermore, other factors including olderage, hypermature cataract, smaller pupils, long axial lengths and pseudoexfoliation increase the risks of posterior capsular rupture. 19,69 Although vitreous opacity is a common post-surgery ocular complication that impairs vision and reduces contrast sensitivity (CS) owing to the presence of lens epithelial cells in the capsular bag after cataract surgery, this was observed in only one participant in this study.^69^,^70^,^71^,^72^

Strengths of this study included the use of a post-surgery six-week follow up period that is consistent with other studies and standardised measurement protocols during the follow up periods. The VA was measured with both the illiterate and literate versions of the LogMAR chart to minimise measurement error.^73^,^74^ The sample also included an almost equal number of participants in the ECCE and MSICS groups to facilitate comparison of the outcomes. Possible limitations of this study include the sample consisted of mainly African (n = 100) and olderparticipants. The absence of near VA, stereopsis, color vision and CS in the assessment of outcomes. Absence of pre-surgery and post-surgery keratometry for comparison of possible surgery induced astigmatism.

## Conclusion

The results of this study are important for the clinical management of patients undergoing the ECCE and MSICS techniques. In developing countries with human resource and equipment challenges, there is a need for low cost yet effective cataract surgery techniques.^16^,^17^ The ECCE and MSICS techniques are performed to restore vision and enable previously visual impaired individuals to restart work and contribute to household income and engage in other activities.^36^ Therefore, it is essential to monitor post-surgery outcomes because it will help further improve expected outcomes^82^ as they are of significant importance for the WHO “Vision 2020” programme.^20^ The results of this study demonstrated that the MSICS technique shows a significant clinical improvement in post-surgery aided VA, less post-surgery refractive astigmatism and is associated with few post-surgery ocular complications compared with ECCE technique. Therefore, wherever the requisite surgical expertise and physical resources are available, the MSICS technique is recommended as the technique of choice for optimal outcomes post-surgery in developing countries.^19^,^38^
